# CD8+ T cell responses in metastatic melanoma patients receiving an adenovirally antigen engineered dendritic cell vaccine +/- IFN-α

**DOI:** 10.1186/2051-1426-3-S2-P149

**Published:** 2015-11-04

**Authors:** Samuel Du, Patricia M Santos, Hussein Tawbi, Ahmad Tarhini, John M Kirkwood, Lisa H Butterfield

**Affiliations:** 1 Pomona College, Claremont, CA, USA; 2 University of Pittsburgh, Pittsburgh, PA, USA; 3 University of Pittsburgh Cancer Institute, Pittsburgh, PA, USA; 4 University of Pittsburgh School of Medicine, Pittsburgh, PA, USA

## 

Dendritic cells (DC), the primary antigen presenting cells and stimulators of naïve immune cells, are uniquely positioned to promote anti-tumor immunity. We developed a DC vaccine which expresses three full length melanoma antigens tyrosinase, MART-1, and MAGE-A6 engineered with an Ad type 5 adenovirus “AdVTMM2” which can activate CD8^+^ and CD4^+^ T cells as well as natural killer (NK) cells. A clinical trial testing this vaccine as well as the potential effects of IFN-α administration post-vaccination has enrolled 36 patients to date (NCT01366144). Peripheral blood banked at baseline, post-DC vaccination, and after either observation or one month of high dose IFN-α was tested for anti-tumor immunity. Here, we present initial immune response testing of the 12 HLA-A2^+^ patients who were able to be assessed for circulating CD8^+^ T cell frequencies by HLA-A2-peptide dextramers. Patient PBMCs were analyzed by MHC dextramer binding assay to determine 1) the frequency of CD8^+^ cells specific to vaccine encoded antigens in the subset of HLA-A2^+^ patients and 2) potential determinant spreading to antigens not in the vaccine, 3) frequency and co-expression of the checkpoint inhibitor molecules CTLA-4, PD-1, and TIM-3 on CD8^+^ T cells, and 4) to characterize three NK cell subpopulations. On the CD8^+^ T cells, PD-1 was the checkpoint molecule most commonly expressed, while CTLA-4 was minimally expressed. TIM-3 was the checkpoint molecule most commonly expressed on all three subpopulations of NK cells. We observed that most patients developed vaccine-encoded antigen-specific responses, and a subset demonstrated determinant spreading to non-vaccine encoded antigens gp100 and/or NY-ESO-1. Expression of checkpoint molecules changed on both T and NK cells through the treatment periods, and the function (by IFNγ ELISPOT) was also assessed. This study will aid in the design of more effective dendritic cell vaccines and adjuvants for metastatic melanoma patients.

## Trial registration

ClinicalTrials.gov identifier NCT01366144.

